# Review of the State of the Art of Deep Learning for Plant Diseases: A Broad Analysis and Discussion

**DOI:** 10.3390/plants9101302

**Published:** 2020-10-01

**Authors:** Reem Ibrahim Hasan, Suhaila Mohd Yusuf, Laith Alzubaidi

**Affiliations:** 1 School of Computing, Faculty of Engineering, Universiti Teknologi Malaysia, Skudai, Johor 81310, Malaysia; ihreem@graduate.utm.my (R.I.H.); suhailamy@utm.my (S.M.Y.); 2 Al-Nidhal Campus, University of Information Technology & Communications, Baghdad 00964, Iraq; 3 Faculty of Science & Engineering, Queensland University of Technology, Brisbane, QLD 4000, Australia

**Keywords:** plant diseases, shallow classifier, deep learning, transfer learning, feature visualisation, feature extraction

## Abstract

Deep learning (DL) represents the golden era in the machine learning (ML) domain, and it has gradually become the leading approach in many fields. It is currently playing a vital role in the early detection and classification of plant diseases. The use of ML techniques in this field is viewed as having brought considerable improvement in cultivation productivity sectors, particularly with the recent emergence of DL, which seems to have increased accuracy levels. Recently, many DL architectures have been implemented accompanying visualisation techniques that are essential for determining symptoms and classifying plant diseases. This review investigates and analyses the most recent methods, developed over three years leading up to 2020, for training, augmentation, feature fusion and extraction, recognising and counting crops, and detecting plant diseases, including how these methods can be harnessed to feed deep classifiers and their effects on classifier accuracy.

## 1. Introduction

**Figure 1:**
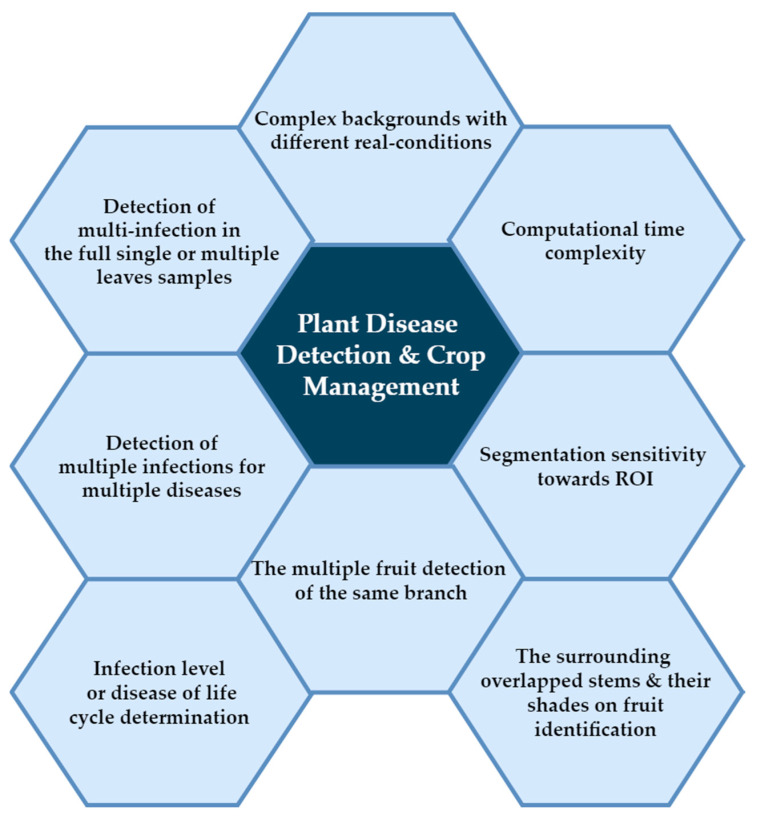
The current challenges of plant disease detection and crop management.

Currently, there are widespread applications of deep learning (DL) around the world. These applications include health care [1], visual data processing [2], social network analysis [3], and audio and speech processing (e.g., recognition and enhancement) [4]. The efficacy of DL models such as reinforcement learning [5], long short-term memory [6] and auto-encoders [7] for solving dimensionality problems has been proven. In contrast with previous machine learning (ML) algorithms that employed several analytical measures for feature extraction, DL techniques learn features directly and represents them successively in the hierarchical architectures [8]. Deep learning technology has been successfully employed as a robust tool in image classification [9] and disease detection based on medical images in the biomedical field [10,11]. The use of DL has also been investigated in the agricultural and plant disease field [12] to support better agriculture [13] and improve the quality of crop management [14]. Losses in the functions in the agrarian sector can affect the economy of countries that rely mainly on this industry [15]. Many factors may cause these losses; these factors can be abiotic or biotic [16,17,18]. There are some limitations [19,20] that are still considered as challenges for researchers using unsupervised models. These challenges are highlighted in (Figure 1) and discussed in this section.

One of the current problems with unsupervised disease detection models is time complexity. However, the high achieved accuracy of k-mean clustering algorithms [21] is still in need of more computational time concerning the index validity term. Another key problem is the segmentation sensitivity towards the region of interest (ROI) determination [22,23,24]. Researchers have attempted to hybridise unsupervised models with specific statistical measures [25,26] or artificial intelligence algorithms to enhance their performance. There has also been a tendency towards using DL models to solve more complex problems concerning real-time disease detection in the fields. One study [27] achieved 98.42% accuracy for the overall dataset by applying a convolutional neural network (CNN) based on the GoogLeNet architecture for apple-leaf disease detection based on a unified background plant village dataset. Another study [28] proposed a new model, based on MobileNet, that depends upon two convolutional steps. First, a depthwise separable convolution is implemented at a single convolution depth slice. Second, pointwise convolution integrates information via the whole depth. The objective of this new architecture was to deploy a mobile application that decreases the computational latency issue with six times fewer parameters than standard MobileNet.

Using a plant village dataset exclusively [29], high accuracy can be obtained only by training models using real-condition samples. The detection of multiple infections in single or multiple leaves is another challenge in this field. One study [30] proposed two CNN models: one trained using full leaf sample images, and the second trained using segmented leaf samples containing different symptoms from the same training dataset of the first model. The findings indicate that the second model showed superior performance over the first model based on measurements of both final classification accuracy and the Quartile Coefficient of Dispersion (QCoD) of confidence difference between the models. However, it was unable to detect multiple infections for multiple diseases, and improvements are needed at the level of segmentation. Other issues include the detection of small objects for multi-label image datasets, pest detection [31,32], infection level determination, disease life cycle identification and mild symptoms [33]. Another challenge in this domain is fruit recognition for harvest purposes; the authors of [34] proposed an altered Yolo architecture, comprising 11 layers, that divides the input image into two grids of small blocks to improve detection performance. According to the researcher, the developed method could not detect more than one object in the same grid cell. The identification process in classifiers is based on how ROIs are localised. Many techniques have been discussed in relation to this issue, including segmentation, object detection and hybrid methods that supply classifiers with contextual information related to the ROI and thereby affect their performance [35].

The present paper aims to investigate the current research orientations in plant disease detection. The contributions of the study are as follows:

- Detailed investigation regarding the architectures of recent shallow classifiers and the accompanying handcrafted techniques for handling features.
- Discussion of recent deep classifiers and the accompanying enhancement techniques for handling features as well as the effects of transfer learning and additional contextual information on the accuracy of these classifiers.
- Description of recent investigations regarding publicly available plant datasets and discussion of the architectures that are used for data augmentation.

## 2. Deep Learning Challenges

Deep learning has achieved impressive performance in several tasks including visual recognition, language, and speech detection systems, besides drawing attention to its research sites and considerable advances [36,37,38]. Conversely, due to the lack of general public data availability and their challenging nature, numerous fields are almost have not considered yet by DNNs. Thus, it is a fertile ground and generates important opportunities for compensating upcoming research opportunities. This section discusses some of the main deep learning challenges and possible solutions including the hardware options. The detection and classification of plant diseases with deep learning are facing the same issues.

The lack of adequate training samples with labels is the most challenging issue of the deep learning tasks [39]. Each day petabytes of data are included, excluding the zettabytes of presently existing. This huge progress is heaping up the data, which could not be labeled without human help. The present success of supervised learning techniques is typically due to the current large datasets and the instantly existing labels [40]. Conversely, unsupervised learning techniques will become the main considered solution with a quick rise in data complexity and size [41]. In addition, capturing the approximated information throughout observations instead of training is the way that the emerging issues like messy data, missing data, and data sparsity, enforce the current deep learning models to be revised. Further, low-sample, unlabeled, high-dimensional, heterogeneous, and incomplete datasets are unlocked sites for deep learning techniques. This is an extremely motivating since the exclusive capability in working with the unsupervised data is provided due to the inbuilt doubting black-box nature of the DNN [42]. Numerous enhanced deep learning models are developed for handling messy and noisy data [43]. Eighty-million undersized images were undertaken the challenging database, which comprises low-resolution RGB photos using 79,000 inquiries, as in Torralba et al. [44]. To decrease the noisy labels in the data, they employed a new robust cost function. Further, massive volumes of data in a streaming-live format are used in several applications nowadays such as social networks, XML files, DNA/RNA sequencing, and time series. These volumes of data suffering the problem of unbalanced, heterogeneity, and incomplete data. Currently, it is a relevant problem and still under discussion on how deep learning models learn in such fields.

Vanishing and exploding Gradients are other significant challenges for deep-learning techniques. The inherent approach of the deep learning network means that each layer computes derivatives (or gradients) in a way of cascading (layer by layer). In addition, derivatives are exponentially increased or decreased in each layer. This approach is disposed to exploding (or vanishing) gradients. However, weights are decreased/increased, depending on gradients, to decrease the error or the cost function. The network could consume an extended time for training due to extremely small gradients, while the training may diverge or overshoot due to large gradients. This issue becomes worse, using the non-linear activation functions such as tanh and sigmoid functions, which compress the outputs to a narrow span. In addition, any weight variation has an insignificant impact on the output training and may consume extra extensive time. Linear activation functions such as proper weight normalization and ReLU can reduce the effect of this problem.

Overfitting is one of the most common issues of deep learning models. Adding extra neurons to a deep learning network leads certainly to form the network for extra-complicated issues. The deep learning network has the ability to provide itself with great adaptability to the process of data training. In contrast, there is an additional chance of overfitting risk to the noise and outliers during the data training process. This issue causes a delay in times of training and testing, as well as, lowers the prediction quality on the real test-data. For instance, in problems related to cluster or classification, overfitting generates a high-order polynomial output. This output isolates the determination limit for the training dataset, which consumes extra time and lowers the outcomes for nearly all the test dataset. Choosing wisely several neurons in the hidden layer for matching the problem type and size is one way to solve the overfitting issue. Various algorithms are available for approximating the proper number of neurons. Unfortunately, no magic bullet is there, but the best answer is experimenting with each use-case to obtain the optimum value.

Lastly, utilizing the minimum volume of resources while completing the maximum throughput is another issue of deep learning interacted with the computational efficiency issue [45]. Approaching the up-to-date performances needs significant volumes of computational resources for the present deep learning frameworks. The employment of reservoir computing is one technique that tries to overwhelm this challenge. Utilizing the incremental approaches is another alternative, which uses large and medium datasets with offline training [46]. Currently, several researchers have modified their ideas to implement scalable and parallel deep-learning frameworks [47,48]. Most recently, their idea is further modified towards transfer the learning task on GPUs. GPUs are infamous related to their leakage currents, which in turn, abstracting every reasonable fulfillment of deep-learning models on moveable devices [49]. Employing FPGAs (Field-Programmable-Gate-Arrays) is another solution [50]. FPGAs have been used as deep-learning accelerators for optimizing the data-access pipelines to accomplish considerably enhanced outcomes [51]. A scalable architecture known as DLAU (Deep Learning Accelerator Unit) was used by Wang et al. [52]. The DLAU utilizes three pipelined processing units. As compared to CPUs, they achieved 36.1 times faster with a consuming power of 234 mW using the locality and tile techniques. Another approach achieved a detection rate of 97%, which utilized an architecture founded on low-end FPGAs with leakages, arc losses, and “so forth and still manages”. As compared to software implementation, they accomplished a proceeding speed of 7.5 times faster. The FPGAs can be merged on a motherboard and they consume less power than GPUs for the same performance output. In contrast, the GPUs offer peak floating-point performance. Zhang et al. [53] proposed a unique approach for implementing CNNs based on a roofline model. They employed the loop tiling to determine the required memory bandwidth, which is a critical issue in the FPGAs. Their model considerably decreases the consumed power and they accomplished 61.62 gigaflops under 100 MHz. Note that a gigaflop is a measuring unit for the performance of the floating-point processing unit. Unluckily, no FPGA testbeds based deep learning is there currently. Therefore, the exploration of this subject is limited just for the experts in the FPGA design.

## 3. Feature Representation in Shallow Classifiers

In cases where the dataset is limited, shallow classifiers are used. These depend mainly on several phases that help in feature extraction and include Support Vector Machine (SVM) classifiers [54,55], random forest [56] and K-nearest neighbours [57,58].

### 3.1. Analysis Measures

Feature texture analysis techniques help in pre-determining the characteristics (shape, distance, colour space, location) of the ROI in the input image. They are first presented as 14 Haralick features [59] and then extended to 18 features [60]. The Grey-Level Co-occurrence Matrix (GLCM) technique is used for greyscale global feature extraction. The Binary Quaternion-Moment-Preserving (BQMP) [61] technique is used for colour edge detection, multiclass clustering of colour and colour feature analysis; it represents parts of an image with specific colours existence using a histogram. The Histogram of Gradients (HOG) model can be used for fast feature appearance extraction [62]. A spatial grey-level dependence matrix can indicate THE orientation, distance, location and size of an ROI [24,63]. Auto-Regression (AR) has a degree of randomness that enables prediction of the colour cell based on the data of the previous cell. The Markov random field model measures the probabilities of the joint cells in the ROI. Recently, the colour co-occurrence matrix has been proposed for local optimisation [64]; this technique depends on the visible spectrum of the coloured features, and it provides more characteristics than GLCM.

### 3.2. Segmentation

These features are represented in vectors that undergo segmentation techniques based on lesions [65], Otsu [66], ROIs [67] or edges [68] to separate ROIs from the background. This is followed by a fusion process to combine the feature vectors in a final vector. Thus, the classifiers can categorise the region of infection in an input image sample by comparing it with the final vector of the combined features. Many fusing techniques are used, including wavelet transformation [69] to decrease noise and discriminant correlation analysis [70] to maximise pair-wise correlation. Additionally, a global–local method can be used for coloured feature extraction, as presented in [71], where the features concatenate to a single vector. However, this concatenation may increase the features of the vectors’ dimensionality, leading to increased complexity. To address this problem, supervised Principal Component Analysis (supervised PCA) with a covariance matrix was used in [72] to retain the high covariance of the original data and to differentiate the features of interest. Additionally, the study used feature selection to reduce redundancy by selecting only the relevant features; then, functional fusion was applied rather than concatenation to feed the Support Vector Machine (SVM) classifier, proving its stability and accuracy up to 90%.

In [73], an approach of feature extraction and fusion by canonical correlation analysis was proposed, along with neighbourhood component analysis to reduce irrelevant features. Then, features were then fed to an M-class SVM, which achieved 92.75% as accuracy. Other methods, e.g., genetic algorithms (GA) and Particle Swarm Optimisation (PSO), have also been proposed for lesion segmentation [24].

**Figure 2:**
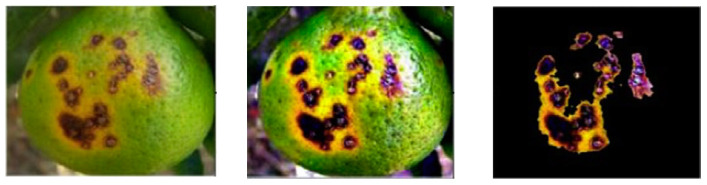
Citrus fruit in a real environment with the infected region of the real image highlighted for map segmentation purposes [25].

A detection method that achieved 96.90% accuracy was proposed in [25]. It combined several processes: first, the original image was enhanced using a hybrid Top Hat–Gaussian filter to illuminate the undesirable effects of brightness; second, the infected regions were highlighted (as in Figure 2) to differentiate them from the background by using a threshold skewness; and finally, a weighted High-Dimensional Colour Transform (HDCT)-based saliency segmentation was applied, proving its efficiency compared with Otsu, Expectation Maximisation (EM) and contour segmentation.

**Figure 3:**
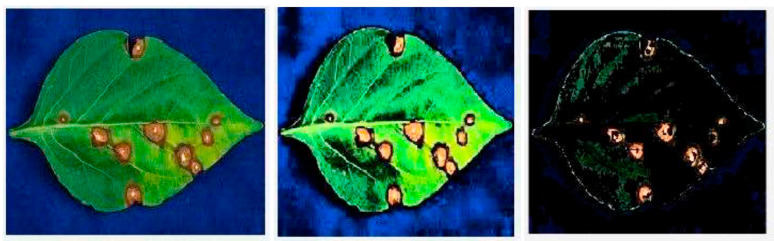
Capsicum leaf segmented, with infected regions highlighted; 100% accuracy achieved for Support Vector Machine (SVM) and K-Nearest Neighbors (KNN) classifiers [26].

Histogram equalisation was applied to a Capsicum dataset combining 70 images of leaves and fruits taken with a high-resolution camera [26] to enhance the image contrast and highlight the regions of infection, as shown in (Figure 3). The enhancement phase facilitated the texture feature extraction phase by using the GLCM technique, which achieves a 100% accuracy level when it feeds the classifier. These features are quite important for the use of SVN classification levels, which are as follows: the contrast of intensity among contiguous pixels upon the image, the correlation of contiguous pixels upon the image, the entropy (the clutter of intensity in a region), the energy of pixels’ uniformity, and the homogeneity of similar pixels [26,74].

**Figure 4:**
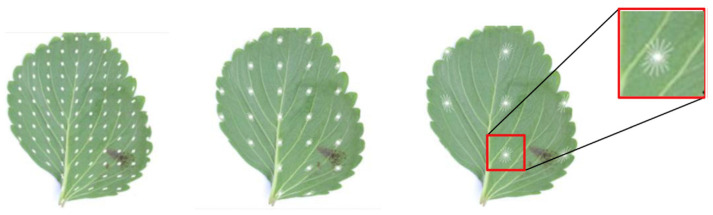
Strawberry leaf with different cell sizes Histogram of Gradients (HOG) was applied (white arrows indicate the obtained gradient information; RGB patches directions and arrow lengths represent the size of the gradients) [75].

A dataset containing 254 strawberry healthy/diseased leaves was used in [75]. Image samples were obtained from fields, and the HOG technique was applied to provide information about gradient (size and direction), as shown in (Figure 4), that is suitable for determining the shape and the orientation of the leaf. Accelerated or Speed Up Robust Features (SURF) and GLCM techniques were applied to establish the key point feature to feed to an Artificial Neural Network (ANN) classifier; the model achieved 94.34% accuracy.

## 4. Evaluation Measures

There are statistical evaluation measurements were used for analyzing the quantitative performance of the classification models including the detection and classification of plant diseases with deep learning. These measurements classify the image samples into four statuses as follow: TP is the number of true-positive image samples that are perfectly identified as infected, FP represents the number of false-positive image samples that are wrong classified as infected. TN is the true-negative image samples that are correctly classified as healthy. FN is the false-negative image samples that are wrongly identified as non-infected. FP is the false-positive image samples that are wrongly identified as infected. These statistical measures are classified as follow:

- Sensitivity /recall: is the ratio of the true-positive samples to all infected samples (true-positive and false-negative). This measure is used to evaluate the performance of a proposed model in predicting true-positive cases [76,77].

(1) $$\mathrm{Sensitivity}\;=\;\frac{\mathrm{TP}}{\mathrm{TP}+\mathrm{FN}}$$

- Specificity: is the ratio of the true-negative samples to all healthy samples (true-negative and false-positive). This measure is used to evaluate the performance of a proposed model in predicting true-negative cases.

(2) $$\mathrm{Specificity}=\frac{\mathrm{TN}}{\mathrm{TN}+\mathrm{FP}}$$

- Accuracy: is the ratio of the correctly classified samples to the total number of classified samples. this measure is used to evaluate the overall performance of a proposed model.

(3) $$\mathrm{Accuracy}=\frac{\mathrm{TP}+\mathrm{TN}}{\;\mathrm{TP}+\mathrm{TN}+\mathrm{FP}+\mathrm{FN}}$$

- Precision/positive predictive value (PPV): is the ratio of the correctly classified samples as infected to all the identified samples as infected (TP+FP).

(4) $$\mathrm{Precision}=\frac{\mathrm{TP}}{\mathrm{TP}+\mathrm{FP}}$$

- F1 Score: is the consistency mean of sensitivity and precision, in the case where the imbalance of false positive/negative samples is important to be measured.

(5) $$F1\;\mathrm{Score}=\frac{2\times \left(\mathrm{Sensitivity}\;\times \;\mathrm{Precision}\right)}{\left(\mathrm{Sensitivity}\;+\;\mathrm{Precision}\right)}$$

- Coefficient of Quartile Deviation: measures the variability of among the image samples themselves and around the average. low coefficient value means low dispersion. Whereas, *Q*_3_ represents the observations that have upper quartile, *Q*_1_ represents the observations that have lower quartile [30].

(6) $$\mathrm{QCoD}=\frac{(Q_{3}-Q_{1)}}{\left(Q_{3}+Q_{1}\right)}$$

## 5. Feature Representation in Deep Classifiers

In cases where the dataset is small, or vast, or varied with a complex background, DL classifiers are used. With this type of classifier, there is no need for the manual techniques.

### 5.1. Training and Transfer Learning

**Figure 5:**
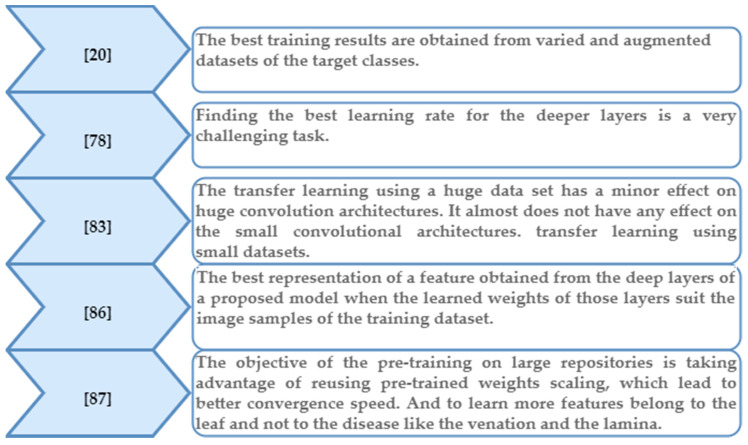
Basic findings regarding transfer learning [20,78,83,86,87].

To train a DL classifier, large datasets are required, comprising a vast number of parameters to be tuned to control training convergence. All the parameters of the proposed model have to be managed (bias, weights, learning rate, mini-batch size, epochs) according to random Gaussian distributions, which is considered a very challenging task [78]. Learning or training from scratch consumes data and time. The availability of large, multi-labelled and well-annotated dataset repositories eliminates the need for researchers to collect massive datasets in different real conditions and environments that would need the oversight of agricultural specialists to be interpreted. Transfer learning allows a CNN model to acquire weights from another model that has already been pre-trained on a large labelled dataset [79,80,81]. The pre-trained model’s parameters must be fine-tuned, and the final layer is replaced with a new layer for convenient transfer of the weights to the proposed new model and the new classes in the target dataset. In [78], all the layers of a CNN model were fine-tuned using the ImageNet dataset at a specific learning rate that was lower than the default rate. The last fully connected layer was randomly initialised and trained to be adequate for the new classes; however, according to the researcher, finding the best learning rate for the other, deeper layers was a very challenging task. Another study about the pre-training effect on plant disease classifiers [82] compared the usage of nine different DL models by employing two approaches: first, transfer learning with the replacement of the last three layers with (fully connect, SoftMax, classifier) layers; and second, taking the result of the feature extraction at specific layers of these models and feeding them to different machine learning (ML) classifiers. The second approach was faster and achieved higher accuracy than the first. The highest accuracy levels achieved were 97.45%, resulting from the feature extraction of ResNet101 with an Extreme Learning Machine (ELM) classifier, and 97.86 from ResNet-50 with an SVM classifier. Meanwhile, transfer learning using small datasets with ResNet-50 achieved 94.60% accuracy [83]. In [20], the proposed transfer learning strategy was enriched by using three different datasets: first, the PlantVillage repository, which was further labelled with two bounding boxes to determine the leaf and infected areas; second, a collected dataset containing certain mandatory information (e.g., a certain disease’s life cycle for a specific plant for certain species); and third, an artificially generated dataset. The accuracy obtained by the trained model was 93.67%. In [84,85], the researchers suggested a first round of training using transfer learning from the original and augmented samples of a large-scale dataset which is in the same domain of the targeted dataset, followed by re-training the model using the original and augmented samples of the target dataset. The highest accuracy achieved was 97.40%. The basic findings of transfer learning are listed in Figure 5.

### 5.2. Feature Visualisation

Feature visualisation methods facilitate the evaluation of classifiers’ behaviour towards the region of infection; every pixel in the selected area has an impact on the activation function. Visualisation techniques observe whether the classifier has selected all the areas of disease in the given image without being affected by the background or noise. In [35,88], the occlusion technique was shown to have an issue in identifying whether a pixel represented symptoms of the right class or part of the background. Additionally, in [80], it was found that the occlusion technique was time-consuming.

The Gradient-weighted Class Activation Mapping (Grad-CAM) algorithm was applied in [33], which had previously been proposed by [89]; it averages the gradient of the objective class on all feature maps to assess their degree of importance. Then, it generates a heat map to represent the ROI. According to the findings in [90,91], this heat map does not provide a full visualisation for the target feature. The heat map produced by faster-R-CNN provides an overall visualisation for the feature, including the centre point and the boundaries; however, as explained by [33], the similarity of symptoms that indicate certain diseases at some stages leads to misclassification. This can be handled by enlarging the training dataset that feeds the Grad-CAM algorithm, and the heat map only highlights the learned features. In [79], the researcher proposed applying the guided back-propagation technique alongside the saliency map technique; the efficiency of this approach was proven, with the results highlighting all the spots of infection [92]. In [93], various visualising methods were tested in every layer of a CNN to determine the suitable layer for the visualisation, and a ‘shaving parameters’ method was presented that proved the efficacy of these methods. Hence, the researchers were able to reduce the required parameters/layers by 75% without affecting accuracy. Therefore, the shallow layers were sufficient for CNN classification.

### 5.3. Architectures

Many CNN architectures have been developed recently; according to [94,95], the DenseNet structure [96] can achieve almost the same accuracy level as ResNet [97] with fewer parameters. These models improve their prediction phase by adopting non-CONV layers, residual learning and batch normalisation. Both architectures decreased computational time by reducing the convolution filter sizes, so these filters were smaller than those in the predecessor architectures VGG [98] and AlexNet [9].

RCNNs are two-stage object detection architectures [99]. In the Faster-R-CNN version, the ROI is filtered via the fully convolutional region proposal network then, the filtered features are shared with a detection network called the ROI pooling network; this ensures that the extraction process is fast and accurate [100,101]. However, this method has a quantisation problem that affects ROI prediction. Thus, the Mask R-CNN used in [102], which is an updated Faster-R-CNN model, was thus presented with a ResNet–feature pyramid network (FPN) backbone. The FPN enabled small object detection and enhanced semantic segmentation. Additionally, the ROI pooling was replaced with pixel-to-pixel alignment; all the computational values of the features were taken into consideration.

In contrast, YOLOs are one-stage object detection architectures. Yolo-v3 [103] was developed as a real-time architecture for predicting multiple classes from an input image without any pre-determination process. It replaced the SoftMax activation function with a cross-entropy function, enabling multiple class detection; it was based on a system similar to FPN, which made its feature extraction robust. Finally, it was faster than Single Shot Detector (SSD) due to employing DarkNet-53 as a backbone. The performance of RetinaNet [104] was shown to be somewhat more practical than Yolo-v3. It harnessed an FPN as a backbone classifier, which re-sharpened the cross-entropy performance by adding a tuneable factor that decreased missed classified cases in training.

In recent years, many DL architectures have been proposed, inspired by standard DL models, to develop ROI determination and disease identification phases, as presented in detail in Table 1.

### 5.4. Concatenation of Additional Information

For a better disease determination, the surrounding climate of the plant could be taken into considerations by combining the result of the fully connected layer with additional information (e.g., environmental or geographical).

The abstraction-level fusion algorithm for framing is a CNN that was applied on almost 300 image samples of olive leaf (mix of healthy and infected with abiotic/non-abiotic factors) [105]. It supplies the fully connected layers of the network with additional features obtained from three different baseline architectures. This step enhances the features represented in the successive layers. Hence, for the appearance of remarkable features in unhealthy leaf segmentation maps, the achieved accuracy was 98.60%. The abstraction of layers increased with increasing levels of complexity. The multi-context fusion network [106] was trained and tested using more than 50,000 image samples across 77 categories. It was employed to concatenate contextual and visual information. The contextual information concerned the environmental factors surrounding the plant (e.g., humidity and temperature), which may cause or lead to specific diseases. The categorisation of these factors improved the identification phase, where 97.50% accuracy was achieved. However, dataset limitations are an issue due to the difficulty of collecting image samples covering the massive range of crops in different environmental conditions. The Deepest model [31] was applied to detect pests in crops; it was able to identify tiny features such as pests with an inference time of 120 ms. The model utilised a detection process cascade. First, a two-level DecisionNet was learned by using contextual information extracted from crop images as prior knowledge for the classifier. These images consisted of different species infected with pests, and the objective of this net was to detect the category of the crop. Second, small-scale information projections occurred in high convolutional layers, with a DeepPest model employed to detect small pest features. An accuracy of 90.70% was achieved.

**Figure 6:**
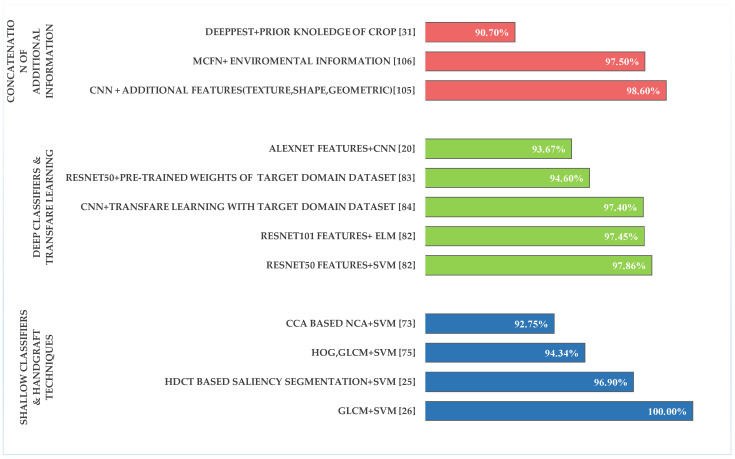
Highest accuracy levels achieved using different techniques.

These methods has been compared in (Figure 6) with different types of technique that handle features in a way that makes classifiers able to diagnose the target object, Where in refrences [105,106], effective results were achieved in disease identification levels due to the ability of the model to categorise the factors of the symptoms. However the high accuracy achieved using shallow classifiers [25,26] with the handcrafted techniques, deep classifiers are still obtaining [82,84] better accuracy thant shallow ones.

**Table 1:** Analysis of recent studies in crop, pest and disease detection using deep learning (DL) techniques. (Weaknesses = challenges facing researchers; Architecture strength = how these challenges are handled; and Findings = the results of the enhancements.).

Fruit Detection Architectures and Plant Disease Detection Architectures				
Study	Objective	No. of Images	Analysis	Evaluation
[32]	Cucumber detection	255 images containing 218 cucumber fruits	Weaknesses: Difficulty of extracting the fruits from image samples with complex backgrounds and overlapping stems of the same colour. Architecture strength: Multi-path CNN suggested the replacement of the SoftMax with SVM classifier for better feature extraction. ROI analysis based on the transformation of the colour space to 15 colours and the PCA analytical tool. Findings: Decreased feature probability for the SVM classifier (the input image). Despite the obstructions with overlapping stems, which are almost the same colour as the fruits, this method was able to recognise both.	90% correct recognition
[107]	Tomato detection	966 images containing 3465 tomato fruits	Weaknesses: False positive detection in some cases of severe fruit occlusion or overlapping leaves. Architecture strength: YOLO-Tomato utilised a dense architecture inspired by DenseNet. It employed circular boxes rather than rectangular boxes. Findings: The model enabled the re-usage of features at successive layers for better feature extraction, which helped address the gradient vanishing problem by decreasing overlapping and occlusion effects, resulting in improved tomato localisation.	94.58% correct recognition
[108]	Grape detection	300 images containing 4432 grape clusters	Weaknesses: Counting grapes is very challenging due to the variability in shapes, sizes, compactness and colour. Architecture strength: Mask R-CNN with ResNet 101 as a backbone. Findings: Simultaneous localisation and mapping algorithms allowed the model to overcome these challenges and helped to prevent double counting of fruits.	0.91 F1-score
[109]	Branch fruit recognition	12,443 images containing apples, nectarines, apricots, peaches, sour cherries, plums	Weaknesses: In cases of apples overlapping with leaves, the activation map was more sensitive towards the leaves than the fruits. Architecture strength: Composed of three convolutional layers, max-pooling and fully connected layer, and used global average pooling rather than traditional flattening techniques. Findings: Very fast compared to ResNet50, making it appropriate for precision horticulture. Suitable for use with small training datasets.	99.76% accuracy; 0.997 F1-score
[110]	Apple and orange detection	5142 apples in 878 images mix of close-up and distant views	Weaknesses: Without applying any pre-processing, light and shadows of the overlapping leaves impeded the Yolo-v3 architecture’s detection of 90% of the fruits. Architecture strength: Applying pre-processing techniques (contrast increase, slight blurring, thickening of the borders). Findings: Pre-processing increased efficacy.	90.8% detection rate; 0.92 F1-score
[111]	Apple detection	1200 images 100 contained no fruits	Weaknesses: Detection failures in cases of overlapping fruits. The LedNet model was time-consuming. Architecture strength: ResNet model implemented as a backbone. Both FPN and atrous spatial pyramid pooling employed within the model. Findings: The used techniques achieved better ROI detection on the feature map than the convolutional fixed-size kernel used in one-stage architecture.	0.849 F1-score
[112]	Kiwi detection	1000 images – mix of RGB and near-infrared (NIR) modalities, containing 39,678 fruits	Weaknesses: No public kiwi fruit datasets available, presenting an obstacle to comparing the study with different datasets. Architecture strength: The VGG16 network was altered to handle RGB and NIR parameters separately but simultaneously, then fine-tuned. Findings: Fast detection and increased accuracy.	90% accuracy
[113]	Orange detection	200 images	Weaknesses False negatives when distinguishing between fruits and branches. Architecture strength: Mask R-CNN with ResNet-101 as a feature extractor backbone, utilising pixel-wise segmentation. Findings RGB + HVC images enhanced the segmentation phase.	0.9753 precision
[114]	Sugar beet leaf disease detection	155 beet leaf images, 97 containing mild diseases	Weaknesses: High time consumption associated with gradient descent repetition due to the high-resolution image samples and the large volume of stride and padding used in the convolution layers (applied at the training phase). Architecture strength: Updated Faster R-CNN; adjusted the parameters of the model to be suitable for the number of objects in the dataset. The size of the input image, the number of filters, the strides and the padding size increased in the first two convolutional layers. Findings: Provided detailed information about the diseased corner regions of the leaves.	95.48% accuracy
[115]	Detection of insects in stored grain	22,043 images containing 108,486 insects	Weaknesses: False negatives for extremely small-sized insect due to unfit anchor scale contributions that considered these insects as ground truth. Architecture strength: MSI-Detector with FPN backbone; an architecture proposed to extract multi-/small-scale insects surrounded by anchors of corresponding sizes. Included pyramid levels to handle features. Findings: Multi-scale insect detection.	94.77% mAP
[116]	Detection of insects on tea plants	75,222 images of 102 classes (including mites and butterflies) from different datasets	Weaknesses: Spectral residual (SPE) technique highlighted fewer pixels than other techniques. Architecture strength: FusionSUM ensemble network with three different saliency techniques (graph-based visual saliency, cluster-based saliency detection and SPE) applied for feature extraction with different classifiers. Findings: The saliency techniques used improved the performance of all the applied networks; DenseNet and MobileNet were more convenient for large-scale datasets than small-scale ones.	92.43% recognition accuracy
[117]	Banana disease and pest detection (affecting different parts of the plant)	30,952 images containing 9000 leaf images, 14,376 cut fruits, 1427 fruit bunches, 1406 pseudostems	Weaknesses: Many variations experienced by the loss function, especially in the entire plant and leaf models, which had low accuracy compared to the other models. Architecture strength: Faster R-CNN with InceptionV2 as a backbone was the best choice for training, object localisation and increased feature extraction among several architectures that were applied. Findings: This method achieved high accuracy with overlapping dried leaves of the same plant and the surrounding plants.	95% accuracy for pseudostems and fruit bunches
[118]	Tomato disease and pest detection	15,000 images (146,912 labelled)	Weaknesses Time-consuming pyramid architecture. Architecture strength: Improved Yolo-V3, a pyramid architecture and multi-scale images training were applied. The fully connected layer was replaced with a convolutional operation to accommodate the lower number of parameters. Findings: This method improved multi-scale object detection and object dimension identification.	92.39% recognition accuracy
[119]	Apple leaf disease detection	26,377 images categorised into five diseases	Weaknesses: An overfitting problem, perhaps as the wide and deep model selected for better feature extraction had many parameters. Architecture strength: VGG-INCEP; replacement of the first two convolutional layers of VGG16 architecture with others from GoogLeNet Inception for pre-training. SSD-INCEP with Rainbow concatenation employed to fuse features. Findings: This method achieved better feature extraction of different scales and feature fusion.	97.12% recognition accuracy; 78.80% mAP
[120]	Crop disease detection	54,306 images categorised into 14 crop species with 26 diseases	Weaknesses: Essentially depended on the PlantVillage dataset, where image samples have unified backgrounds. Architecture strength: dCrop; an architecture proposed as a mobile application to detect diseases even without internet access. Findings: Three training architectures. The highest accuracy was achieved using ResNet 50, which was able to learn residuals and presented better predictions.	99.24% recognition accuracy
[121]	Rice sheaf and stem disease and pest detection	5320 images of three diseases and 4290 frames of five videos	Weaknesses: Lesion detection in videos is considered very challenging. Hence, the model was trained using still pictures, and avoiding blurry or distorted frames where the boundaries of the lesion are not clear. Architecture strength: DCNN Backbone formed of four blocks, with the ReLU layer in a new position to allow for better convergence. Findings: Many architectures have low detection performance for blurry image samples.	90.0 video spot precision
[122]	Maize leaf disease detection	6267 unmanned aerial vehicle images containing 25,508 lesions	Weaknesses: The large number of sub-images per lesion caused an overfitting problem. Therefore, data were re-split to generate one image per lesion for better training distribution. Architecture strength: Three-stage pipeline model, making full use of the high-resolution image samples. Findings: Adding contextual information in the training phase led to improved putative lesion determination and increased accuracy.	95.1% precision

## 6. Realistic Datasets

One of the difficulties facing researchers is collecting datasets as the accuracy of any DL model can only be improved using large-scale datasets. The PlantVillage dataset, as used in [123], represents the largest open-access repository of crop images. It contains images of both healthy and infected crops with a range of diseases, which are categorised into fungi, bacteria, mould, virus and mites. In this repository, all leaf images are removed from their plants and shown against a grey background, and the samples have been labelled by experts.

Recently, research has revealed that there is a difference in the results of diagnosing models when they are trained with leaf image samples that are removed from the plant and those taken in fields. This difference in results is considered as a drawback in the efficiency of diagnosing models. For this reason, new repositories are available, providing smaller datasets of leaf/fruit images for related/different species. These datasets are available for segmentation, classification and training purposes, and the images for them have been captured in a range of real-life conditions. A publicly available dataset for coffee leaves is named RoCoLe [124]. It contains 1560 images, with a mix of healthy leaves and non-healthy leaves (red mites, several levels of rust). The images for datasets such as this are taken in several environmental conditions, covering a range of different light effects during different times of day and different seasons. The datasets are comprised of images captured with high-resolution cameras, supervised and annotated by professionals, and with backgrounds that include leaves of other plants.

A repository of 12 different plants is provided by [29] mango (healthy, anthracnose), arjun (healthy, leaf spot), alstonia (healthy, foliar galls), guava (healthy, fungal), bael (chlorosis), jamun (healthy, fungal), jatropha (healthy, chlorotic lesions), Pongamia pinnata (healthy, cercospora spot), basil (healthy), pomegranate, lemon (healthy, citrus canker), and chinar (healthy, leaf spot). It combines 4503 sample images of leaves during different stages of their life cycle. Various characteristics of shape, size and colour are observed during the life cycle of a leaf, which can be helpful in further plant health studies. For example, symptoms of abiotic diseases can first appear on a tissue of the leaf.

PlantDoc [125] is a dataset of 2598 multi-leaf images collected from the internet for plant disease detection purposes. It contains 13 plant species: apple (healthy, rust, scab), bell pepper (healthy, leaf spot), blueberry (healthy), cherry (healthy), corn (blight, grey leaf spot, rust), grape (black rot, healthy), peach (healthy), potato (early blight, late blight) raspberry (healthy), soya bean (healthy), squash (powdery mildew), strawberry (healthy), and tomato (bacterial spot, early blight, healthy, late blight, mould, mosaic virus, Septoria leaf spot, yellow virus). However, diseased samples in this dataset are categorised according to APSNET; therefore, some are incorrectly classified due to the lack of professional experience. The PlantDisease dataset [20] contains 18,334 leaf images, covering 12 species: apple, bell pepper, cherry, grape, wheat, onion, peach, potato, plum, strawberry, sugar beet, and tomato. The species are categorised according to 42 classes. This dataset is considered to be the largest leaf repository available. The sample images were obtained under a range of different environmental circumstances.

Small-scale datasets are as an attempt to emphasise or to reveal the efficiency of a proposed plant disease diagnosis method. An online citrus dataset [126] is available that contains 759 sample images of healthy fruits, healthy leaves, non-healthy leaves (black spot, canker, greening, melanoses), and non-healthy fruits (black spot, canker, greening, scab). Another dataset that has recently been made available [21] contains 1000 images of tomato leaves, with a mix of healthy leaves and infected samples (yellow leaf curl, mosaic virus). This dataset includes 200 images with white backgrounds for training purposes and 800 images with natural backgrounds for testing purposes.

## 7. Data Augmentation

Data augmentation is a solution used for solving dataset limitations. Generative adversarial networks (GANs) are a type of model composed of two phases: a generative phase that depicts the data distribution of the input image and a discriminative phase that estimates the probable output sample (the output should be depicted from the training data distribution rather than the first phase) [127]. Efforts have been made to enhance the quality of the created image and training stability for both phases.

Many GAN models have been developed as attempts to overcome the need for large-scale training datasets. Cycle-Consistent GAN [128] employs transitivity for CNN training between two image of two different collections. There is no need for paired training; the mapping strategy depicts high-level features of an image in one domain to the style of another image of another domain, and discriminators and generators are trained symmetrically. This method can be employed to obtain seasonal change effects for the created plant sample or for mapping regions of infection for the constructed plant.

In conditional GANs [127], a new artificial plant image is generated by collecting several masks. These masks represent a specific number of leaves that are added into the synthetic plant image with different sizes and rotation degrees and that represent a real training set distribution.

Several architectures have recently been employed to examine whether the simulated syntactic data in the generated samples have the same characteristics as real samples or not [20]. The Deep Convolutional Generative Adversarial Network (DCGAN) model still showed instability problems in the training phase according to [20,127]. The Progressive growing Generative Adversarial Network (ProGAN) [129] can be used to generate plant leaves by forming small-sized images of 4 × 4 or 8 × 8 pixels up to 64 × 64 pixels to be examined by the discriminator. Then, high-resolution layers are added to the resultant images for training. This model lacks some micro details in features’ structures and so cannot generate an artificial image identical to the real one [20,128].

Autoregressive Deep Convolutional Generative Adversarial Network (AR-GAN) [130] is an approach based on three optimisation functions: the standard GAN, cycle self-consistency, and, to increase the affinity between generated and original images in terms of quality, reconstruction activation [131,132]. The created dataset has been shown to enhance classification by +5.2%.

It is important to note that there are both quantitative and qualitative methods of evaluation for GANs. Average precision is based upon a comparison between the label maps of generated and the real images using semantic segmentation metrics (intersection-over-union per pixel/class).The Fréchet inception distance tool measures the covariance of feature distributions in the real/generated data [133]; the computational efficiency of this method has also been proven for large-scale datasets [130]. Precision measures the quality of created images compared to the corresponding learned dataset, while recall measures the diversity of the generated data [133]. The Neural Image Assessment (NIMA) approach measures the aesthetical and perceptual quality of the artificial image [133,134].

## 8. Discussion

**Figure 7:**
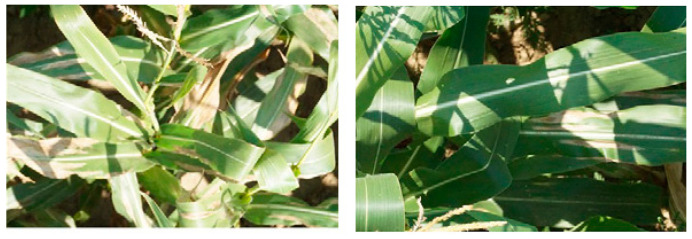
Image samples for lesion spot detection; maize leaves with different natural lighting angles and complex surroundings [122].

**Figure 8:**
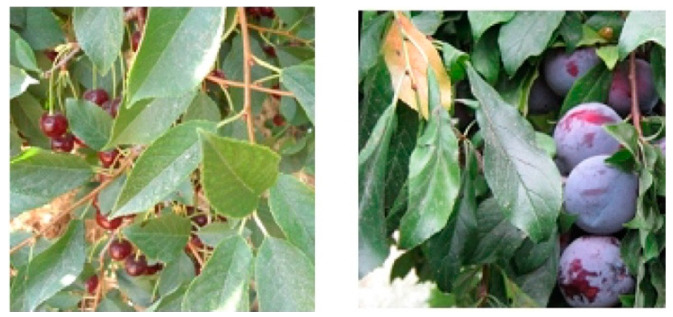
Image samples for fruit detection; cherries and plums with leaf–fruit overlaps [109].

In this section, an analysis of all the architectures discussed above is presented. The major factors that affect deep classifiers are taken into consideration as follows:

- Shallow models are recommended for small datasets. These classifiers can achieve 100% accuracy despite the difficulties of choosing the best analytical techniques to analyse lesion spots and the optimal accompaniment classifier.
- To achieve variety in training image samples, datasets can be collected from different resources. These can take into consideration different natural lighting angles (Figure 7) and conditions with complex overlapping surroundings (Figure 8). This includes the standard augmentation techniques (pixel-wise, rotation, resizing and blurring) and the artificially generated images.

- The impact of target dataset diversity and choosing a backbone model that is suitable for the target classes is much more important than the number of sample images in the dataset itself (as shown in Table 1 [111,113,114]).
- Most of the recently presented models, including YOLO-v3 and R-CNN family architectures, are supported by the FPN model, which enables small object detection and enhances semantic segmentation and multi-object detection (as shown in Table 1 [107,108,110,111,113,114,117,118]).
- Environmental and geographical information has a significant impact on determining whether the symptoms of affected leaves are caused by the surrounding factors or leaf disease.
- The most suitable approaches for determining the efficacy of the pre-training and feature extraction phases of a proposed model are heat-/saliency-map techniques that depict the target objects (as shown in Table 1 [116]).
- Texture and colour analysis techniques and high-resolution image samples are recommended to enhance the feature extraction phase (as shown in Table 1 [32,112,113,119]).
- To validate the accurate performance of a classifier, researchers should test its ability to differentiate similar symptoms related to different diseases (e.g., Northern leaf blight and anthracnose leaf blight in maize leaves). To achieve high accuracy, models should be trained with the target disease dataset [122] and images of similar symptoms on different organs (e.g., withered stems and leaves in rice) [121], as well as the typical viral symptoms in leaves of (melon, zucchini, cucurbit, cucumber, papaya watermelon, cucumber) [33].

## 9. Conclusions and Future Orientations

This review has discussed and analysed contemporary shallow and deep architectures and their highest achieved accuracy levels for plant disease detection and crop management. The use of realistic datasets, augmentation methods and different pre-training backbone models has also been analysed. Despite the successes that have been achieved in this field, there are still some challenges facing researchers and future orientations to be suggested:

- Mild symptoms of some plant diseases in their early life cycles.
- Some lesion spots have no determined shapes.
- Plant health considerations via monitoring growth and ripeness life cycle of fruits and leaves.
- Automated labelling and auto segmentation of image samples based GANs.
- The usage of hyperspectral data to feed deep classifiers is a recently developed technique that is recommended for the early detection of plant disease life cycles and the healthy leaf life cycle to differentiate it from a diseased leaf.
- Lastly, future work will include several deep learning models for early classification and detection of plant disease due to huge improvements in deep learning models and the availability in plant datasets. Therefore, that will reflect positively on the quality of plants for future generations.
